# Identification of plasma hsa_circ_0008673 expression as a potential biomarker and tumor regulator of breast cancer

**DOI:** 10.1002/jcla.23393

**Published:** 2020-08-18

**Authors:** Youting Hu, Qi Song, Jianguo Zhao, Jian Ruan, Fan He, Xia Yang, Xiaocheng Yu

**Affiliations:** ^1^ Department of Breast and Thyroid Surgery Wuhan Integrated TCM & Western Medicine Hospital Hubei China

**Keywords:** biomarker, breast cancer, hsa_circ_0008673, prognosis

## Abstract

**Objective:**

Cell‐free circular RNAs (circRNAs) are stable and abundantly exist in body fluids. In this study, we aimed to investigate plasma cell‐free circRNAs as a novel class of biomarkers for the diagnosis of breast cancer (BC).

**Methods:**

Differentially expressed circRNAs from 6 normal and 6 BC plasma samples were detected by microarray. Hsa_circ_0008673 was then screened and validated in the plasma of 102 normal and 378 BC samples. A receiver operating characteristic (ROC) curve was used to evaluate the diagnostic value. The correlations between hsa_circ_0008673 expression and demographic characteristics, tumor features, and prognosis were analyzed. The effects of hsa_circ_0008673 on BC cell proliferation and metastasis were also measured.

**Results:**

Of the top ten up‐regulated (hsa_circ_0008673, hsa_circ_0008500, hsa_circ_0005260, hsa_circ_0003423, hsa_circ_0119881, hsa_circ_0000987, hsa_circ_0007386, hsa_circ_0000091, hsa_circ_0016601, and hsa_circ_0008549) and top ten down‐regulated (hsa_circ_0000826, hsa_circ_0072697, hsa_circ_0004587, hsa_circ_0000471, hsa_circ_0007786, hsa_circ_0001417, hsa_circ_0005982, hsa_circ_0001566, hsa_circ_0003823, and hsa_circ_0003823) circRNAs from microarray, hsa_circ_0008673 was the most significantly up‐regulated circRNA in BC, and represented a good diagnostic value. Hsa_circ_0008673 was remarkably down‐regulated after breast mastectomy. Hsa_circ_0008673 expression was associated with larger tumor size, distant metastasis, positive estrogen receptor (ER) status, and positive progesterone receptor (PR) status. Additionally, hsa_circ_0008673 could serve as a prognostic predicator of overall survival (OS) and disease‐specific survival (DSS). Cell assays proved that hsa_circ_0008673 knockdown contributed to inhibition of tumor cell proliferation and migration.

**Conclusion:**

Plasma cell‐free hsa_circ_0008673 was up‐regulated in BC, which was associated with poorer prognosis and promoted tumor proliferation and metastasis. Hsa_circ_0008673 is a promising biomarker for tumor diagnosis and prognostic assessment of BC patients.

## 1 INTRODUCTION

Breast cancer is responsible for 14% of total cancer‐related deaths and is the leading cause of cancer in women.^1^ According to the global statistics of 2018, there are estimated 2 088 849 new breast cancer cases and 626 679 breast cancer deaths in 2018, and the incidence and death rate are about 39.2 and 8.6 per 100 000, respectively.^2^ Although early diagnosis, radical surgery, neoadjuvant/adjuvant therapy, and targeted drug applications have contributed to substantial improvements in the survival rate of breast cancer patients with curative intent, the long‐term mortality rate and high recurrence rate remain urgent clinical problems.^3^ As a result, it is essential to investigate novel therapeutic targets that can increase the recovery rate and promote long‐term survival of breast cancer.

Circular RNAs (circRNAs) are class of RNAs formed by back‐splicing events as loops, and are found in all types of organisms.^4^, ^5^ They differ from long noncoding RNA (lncRNA) and microRNAs (miRNAs) in that they do not have the 5′ and 3′ end structures but represent covalently closed cyclic structures.^5^ With newly developed technologies of high‐throughput sequencing and computational approaches, particularly RNA sequencing, up to 30 000 circRNAs have been identified.^6^ CircRNAs are widely involved in the regulation of human physiology and pathology by three main mechanisms including function as a miRNA sponge, as a protein‐binding molecule, and as a template for translation into polypeptides.^7^, ^8^ It is reasonable to hypothesize that dysregulation of circRNAs may influence the progress of various diseases, including cancer.^9^ Zhang et al have reported that most circRNAs express tissue or developmental stage specificity, and circRNAs are involved in the regulation of a variety of biological activities in cancers.^10^ It is important that the unique construction of circRNAs makes them insensitive to ribonucleases, which indicates that circRNAs can exist in tissues and serum, and as a result could serve as biomarkers for human cancer.^11^ Recently, studies have reported that circRNAs could act as promising biomarkers in numerous types of cancers.^12^, ^13^ Li et al have shown that hsa_circ_0000729 is a potential prognostic biomarker in lung adenocarcinoma.^12^ Hsa_circ_0001445 could regulate the proliferation and migration of hepatocellular cancer and serve as a diagnostic biomarker.^13^ CircRNAs have also been proved to play important roles in breast cancer. For instance, Li et al have reported that circular RNA VRK1 is correlated with favorable prognosis, and could inhibit cell proliferation but promote apoptosis in breast cancer.^14^ CircKDM4C could suppress tumor progression and attenuates doxorubicin resistance by regulating miR‐548p/PBLD axis in breast cancer.^15^ CircRNA hsa_circ_0000519 has been reported to be critical in the pathogenesis of breast cancer and may serve as a future therapeutic biomarker.^16^ However, the functions and mechanisms of circRNAs in breast cancer remain uncertain.

In this study, a novel circRNA hsa_circ_0008673 was firstly screened and analyzed in both normal and breast cancer plasma samples. We found hsa_circ_0008673 was significantly up‐regulated in breast cancer patients and positively correlated with larger tumor size, distant metastasis, positive ER status, and positive PR status. We further found that hsa_circ_0008673 was also a promising marker for distinguishing breast cancer patients from normal volunteers compared with CA153 and CEA. Moreover, the OS and DSS of patients with high expression of hsa_circ_0008673 were significantly shorter compared with the low‐expression group. Cell assay further proved that knockdown of hsa_circ_0008673 resulted in inhibiting the proliferation and metastasis of breast cancer cells. In conclusion, plasma cell‐free hsa_circ_0008673 is a promising biomarker for tumor diagnosis and prognostic assessment of breast cancer patients.

## 2 MATERIALS AND METHOD

### 2.1 Patients and specimens

A total of 378 preoperative breast cancer plasma samples, 30 postoperative breast cancer plasma samples, and 102 normal samples were collected from the Wuhan Integrated TCM & Western Medicine Hospital, from December 2016 to May 2019. The eligible criteria were as follows: (a) diagnosed as primary breast cancer by pathological confirmation; (b) age between 18 and 80 years; (c) completed data of tumor feature and survival; and (d) without neoadjuvant therapies before surgery. Plasma samples were stored at −80°C once collected from patients until use. Written informed consent was obtained from all patients before the start of study. This study was approved by Ethics Committee of Wuhan Integrated TCM & Western Medicine Hospital.

### 2.2 Microarray analysis

CircRNA microarray analysis was performed using Human CircRNA Array v2.1 (CapitalBio Technology). Total RNA was quantified using NanoDrop ND‐1000. The sample preparation and microarray hybridization were performed based on the Arraystar's standard protocols. Briefly, total RNAs were digested with RNase R (Epicentre Technologies) to remove linear RNAs and enrich circular RNAs. Then, the enriched circRNAs were amplified and transcribed into fluorescent cRNA utilizing a random priming method (Arraystar Super RNA Labeling Kit). The labeled cRNAs were hybridized onto the Arraystar Human circRNA Array V2 (8x15K, Arraystar). After washing the slides, the arrays were scanned by the Agilent Scanner G2505C. Agilent Feature Extraction software (version 11.0.1.1) was used to analyze the acquired array images. Quantile normalization and subsequent data processing was performed using the R software limma package. Differentially expressed circRNAs were identified through fold change filtering. Hierarchical clustering was performed to show the distinguishable circRNA expression pattern among samples.

### 2.3 RNA extraction and qRT‐PCR

Total RNA was extracted from breast cancer plasma samples and normal samples using the TRIzol reagent (Invitrogen) according to the manufacturer's protocol. Total RNA from each specimen was quantified, and quality assurance was conducted using a NanoDrop ND2000 spectrophotometer (NanoDrop). Reaction mixture (20 µL) containing 1 µg total RNA was reverse‐transcribed to cDNA using PrimeScript RT‐polymerase (Takara). qRT‐PCR was performed using SYBR Green Premix Ex Taq (Takara Bio) and was monitored using the ABI PRISM 7500 Sequence Detection System (Applied Biosystems, Life Technologies). The relative expression levels of circRNAs were determined by qRT‐PCR. The sequences of the primers used in the qRT‐PCR assay are shown.

Hsa_circ_0008673 F: 5′‐ACCTTGGAACTGTGAGA‐3′;

Hsa_circ_0008673 R: 5′‐GCGAAGAGCAGATAAAT‐3′.

GAPDH F: 5′‐GTCTCCTCTGACTTCAACAGCG‐3′;

GAPDH R: 5′‐ACCACCCTGTTGCTGTAGCCAA‐3′.

The reaction conditions were as follows: 95°C at 5 minutes for a preincubation and 40 cycles of 95°C for 5 seconds, annealing temperature of 60°C for primer pairs for 30 seconds, and 72°C for 20 seconds. RNA levels were normalized using GAPDH as the internal control.

### 2.4 Cell culture

MCF‐10A, MCF‐10AT, MCF‐10CA1A, and MCF‐10CA1H cell lines (ATCC) were cultured in Dulbecco's modified Eagle's medium (DMEM)/F12 (Invitrogen) supplemented with 5% horse serum (Invitrogen), 500 ng/mL hydrocortisone (Sigma‐Aldrich), 100 ng/mL cholera toxin (Sigma‐Aldrich), 10 µg/mL insulin (Invitrogen), and 20 ng/mL epidermal growth factor (EGF, Sigma‐Aldrich). MDA‐MB‐231, MDA‐MB‐468, and MCF‐7 were cultured in DMEM (Invitrogen) supplemented with 10% fetal bovine serum (FBS, Gibco). T47D were cultured in RPMI (Invitrogen) supplemented with 10% FBS with and without insulin. All cells were cultured in a humidified atmosphere containing 5% CO2 at 37°C.

### 2.5 Establishment of stable hsa_circ_0008673 knockdown cell lines

Vectors that stable knockdown (pLKO.1) hsa_circ_0008673 were obtained from Vigene Biosciences and were transfected into MDA‐MB‐231 and MCF‐7 using a Lipofectamine RNAiMAX reagent (Thermo Fisher Scientific, Inc) according to the provided protocol. Cells were then cultured with puromycin to filter the stable cell lines. The short hairpin RNA (shRNA) sequence to hsa_circ_0008673 was as follows:

sh‐1, 5′‐TTCTGCAAAAAAGGTTCATTG‐3′;

Sh‐2, 5′‐AAAAAAGGTTCATTGGAACAG‐3′.

### 2.6 Cell viability assay

Cell viability was determined by a 3‐(4, 5‐dimethylthiazol‐2‐yl)‐2, 5‐diphenyltetrazolium bromide (MTT, Sigma) assay. In brief, cancer cells were seeded in 96‐well plates in culture medium and incubated in 5% CO2 at 37°C. After incubation for the indicated time, 20 µL of MTT (5 mg/mL in PBS) was added into each well and incubated for 4 hours. The supernatants were carefully aspirated, and 100 µL of dimethyl sulfoxide (DMSO) was added to each well. Absorbance values at 490 nm were measured on a Microplate Reader (Bio‐Rad).

### 2.7 Cell cycle assay

Cells were collected, washed twice with 1X PBS, and fixed in 70% ethanol at −20°C. After 24 hours of fixation, cells were incubated with RNase A (Takara Bio, Inc) at 100 µg/mL in 1X PBS for 30 minutes at 37°C. Cells were then stained with propidium iodide (PI; BD Biosciences) at 50 µg/mL for 30 minutes at room temperature. Subsequently, cells were analyzed for DNA content using a BD FACSCalibur™ flow cytometer (BD Biosciences).

### 2.8 Migration and invasion assay

Migration and invasion assays were performed as described previously using the Transwell system (Corning Costar).^17^ In the migration assay, 700 µL of medium with 20% FBS was added to the lower well of each chamber and 1 × 10^5^ cells suspended in serum‐free medium were added to the upper inserts. After incubation for the indicated time, the total number of cells adhering to the lower surface of the membrane was quantified in six representative fields. The invasion assay was performed in the same way as the migration assay except that the membrane was coated with Matrigel (BD Biosciences).

### 2.9 Statistical analysis

Statistical differences between independent groups were calculated by Student's *t* test or one‐way ANOVA test. AUC values, sensitivity, and specificity for cell‐free hsa_circ_104056 were quantified by using receiver operating characteristic (ROC) analysis to assess its diagnostic efficiency in differentiating patients with breast cancer from healthy controls. The optimal cutoff thresholds for diagnosis were obtained by Youden index. Correlation was determined by the Spearman test. Patients were equally divided into two groups based on their relative expression, OS and DSS curves were evaluated by the Kaplan‐Meier method, and the survival differences of patients in subgroups were estimated by the log‐rank test. Factors predicting survival were determined by univariate and multivariate Cox's proportional hazard regression. All graphing and statistical analyses were carried out using SPSS 17.0. *P* < .05 (two‐tailed test) was regarded as significant difference, and data were exhibited as mean ± standard deviation.

## 3 RESULTS

### 3.1 Hsa_circ_0008673 was up‐regulated in breast cancer plasma samples

**Figure 1:**
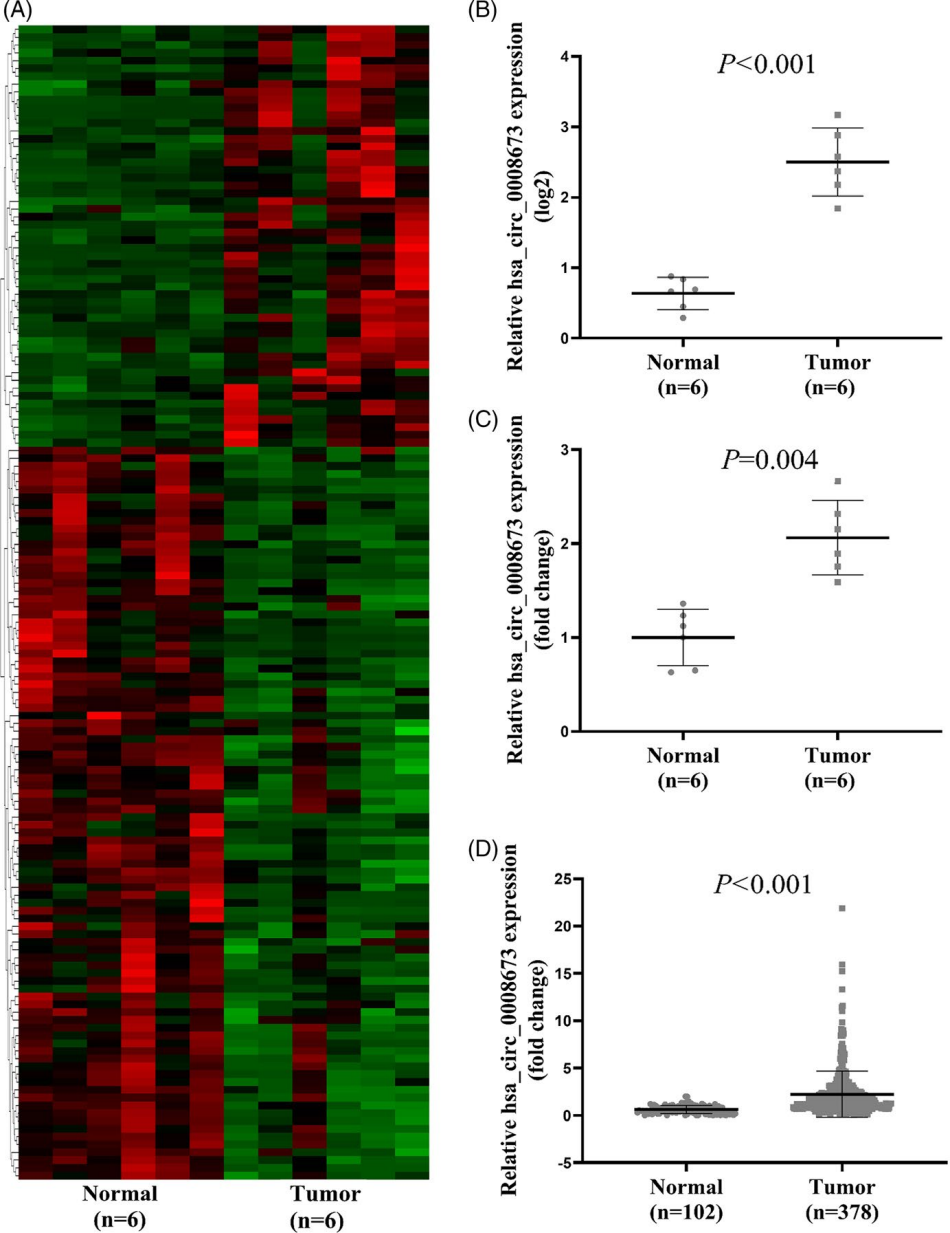
Hsa_circ_0008673 was up‐regulated in breast cancer plasma samples. A, The cluster heat map showed the up‐regulated and down‐regulated circRNAs in breast cancer samples. Red color indicated high expression level, and blue color indicated low expression level. B, The relative expression level of hsa_circ_0008673 in the 6 normal control and 6 breast cancer samples based on microarray. C, The relative expression level of hsa_circ_0008673 in the 6 normal control and 6 breast cancer samples verified by qRT‐PCR. D, The relative expression level of hsa_circ_0008673 in the total 102 normal control and 378 bladder cancer samples

Plasma samples from 6 healthy controls and 6 breast cancer patients were sent for microarray. As shown in Figure 1A, a total of 54 circRNAs were up‐regulated and 94 circRNAs were down‐regulated in breast cancer patients. The detailed information about top ten up‐regulated (hsa_circ_0008673, hsa_circ_0008500, hsa_circ_0005260, hsa_circ_0003423, hsa_circ_0119881, hsa_circ_0000987, hsa_circ_0007386, hsa_circ_0000091, hsa_circ_0016601, and hsa_circ_0008549) and top ten down‐regulated (hsa_circ_0000826, hsa_circ_0072697, hsa_circ_0004587, hsa_circ_0000471, hsa_circ_0007786, hsa_circ_0001417, hsa_circ_0005982, hsa_circ_0001566, hsa_circ_0003823, and hsa_circ_0003823) circRNAs was described in Table S1. Of the significantly changed circRNAs, hsa_circ_0008673 was filtered out because it was the most remarkable up‐regulated circRNAs, and the expression level in microarray is shown in Figure 1B. Moreover, the relative expression between groups was evaluated by qRT‐PCR, which was in accordance with the result of microarray (Figure 1C). The location of hsa_circ_0008673 is chr17: 41247862‐41276132, and the host gene is BRCA1. The genome length is 28 270 bp, and the spliced length is 689 bp. To further evaluate the potential significance of hsa_circ_0008673, a total of 102 normal controls and 378 breast cancer plasma samples were examined by qRT‐PCR. As shown in Figure 1D, hsa_circ_0008673 was remarkably increased in breast cancer patients, which indicates that hsa_circ_0008673 is a potential breast cancer–associated circRNA.

### 3.2 Baseline characteristics and hsa_circ_0008673 expression of the 378 breast cancer patients

The baseline characteristics of the 378 breast cancer patients are shown in Table 1. The average age was 55.48 years, and there were 92 (24.3%) patients in grade I, 254 (67.2%) patients in grade II, and 32 (8.5%) patients in grade III. With respect to T stage, 113 (29.9%) patients were in T1, 228 (60.3%) patients were in T2, and 37 (9.8%) patients were in T3. In regard to N stage, 195 (51.6%) patients showed negative lymph node status and 183 (48.4%) patients were positive. Other detailed baseline characteristics of breast cancer patients are shown in Table 1.

**Table 1:** Plasma cell‐free hsa_circ_0008673 expression and clinicopathologic parameters in breast cancer patients

Variables	Number of cases	Percentage %	Relative expression	*P* Value
Age				
≤60	229	60.6	2.106 (1.760‐2.452)	.906
>60	149	39.4	2.074 (1.664‐2.483)	
Pathological grade (n/%)				
Grade I	92	24.3	2.248 (1.667‐2.830)	.493
Grade II	254	67.2	2.098 (1.772‐2.424)	
Grade III	32	8.5	1.613 (1.076‐2.150)	
Tumor size				
T1	113	29.9	1.705 (1.379‐2.030)	<.001
T2	228	60.3	1.968 (1.619‐2.317)	
T3	37	9.8	4.052 (2.905‐5.200)	
Node stage				
Negative	195	51.6	1.940 (1.628‐2.252)	.237
Positive	183	48.4	2.257 (1.825‐2.689)	
Metastasis stage				
Negative	338	89.4	1.942 (1.679‐2.204)	<.001
Positive	40	10.6	3.376 (2.284‐4.468)	
ER status				
Negative	91	24.1	1.153 (0.846‐1.460)	<.001
Positive	287	75.9	2.392 (2.065‐2.718)	
PR status				
Negative	118	31.2	1.202 (0.860‐1.545)	<.001
Positive	260	68.8	2.498 (2.158‐2.838)	
Her‐2 status				
Negative	321	84.9	2.128 (1.829‐2.426)	.542
Positive	57	15.1	1.900 (1.415‐2.384)	
Radiotherapy				
No	179	47.4	1.949 (1.651‐2.247)	.306
Yes	199	52.6	2.224 (1.800‐2.648)	
Chemotherapy				
No	133	35.2	1.887 (1.523‐2.251)	.257
Yes	254	64.8	2.205 (1.850‐2.561)	

**Figure 2:**
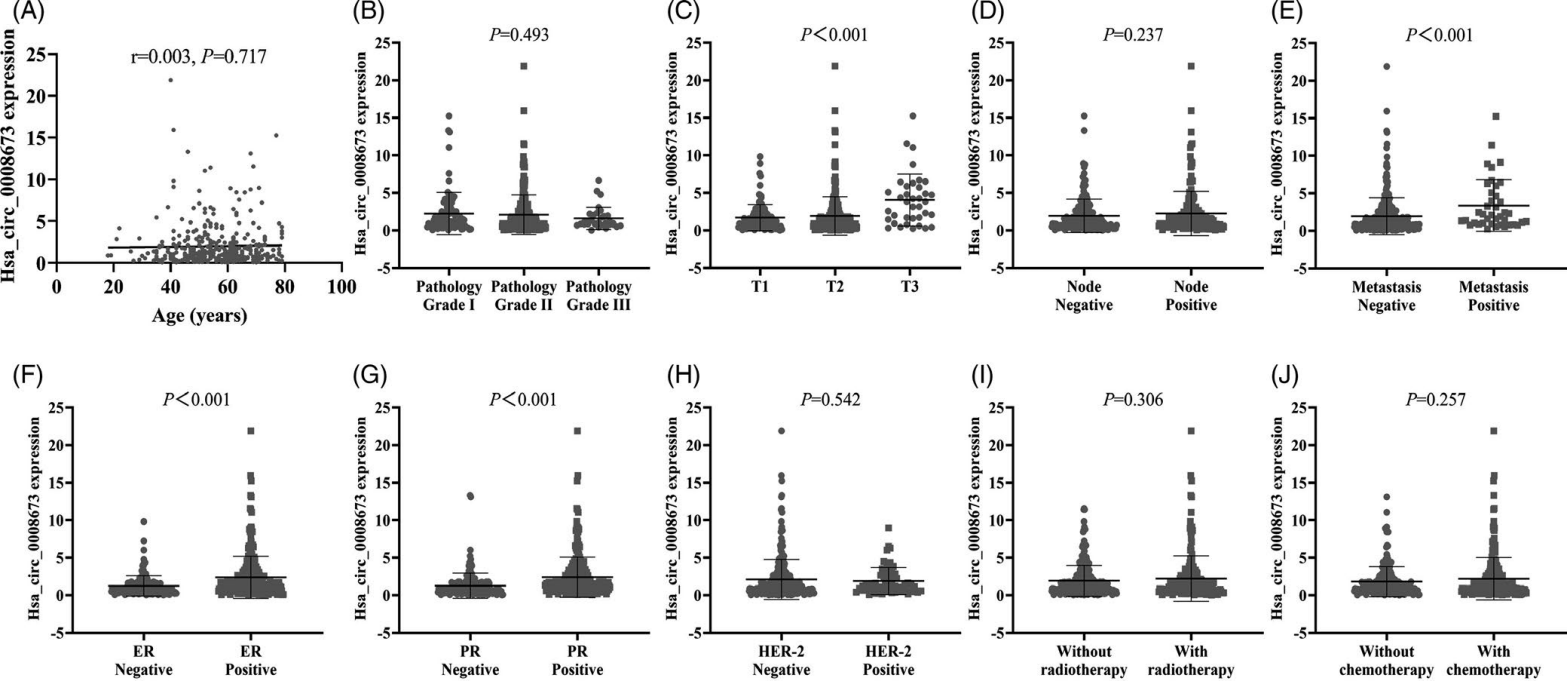
Association between hsa_circ_0008673 relative expression and clinicopathological features in breast cancer patients. Correlation between hsa_circ_0008673 and age (A), pathological grade (B), tumor size (C), lymph node status (D), metastasis status (E), ER status (F), PR status (G), Her‐2 status (H), radiotherapy (I), and chemotherapy (J)

To further investigate whether high expression of hsa_circ_0008673 was related to the clinical progression of breast cancer, we analyzed the expression of hsa_circ_0008673 with the clinicopathological features. The expression of hsa_circ_0008673 showed no significant difference when age, pathological grade, node stage, her‐2 status, radiotherapy, and chemotherapy were analyzed. However, increased expression of hsa_circ_0008673 was found in larger tumor size (*P* < .001), distant metastasis (*P* < .001), ER positive (*P* < .001) and PR positive (*P* < .001). The detailed expression levels among groups are shown in Figure 2. Our results suggest that up‐regulation of hsa_circ_0008673 is associated with malignant biological behaviors of breast cancer, which might influence the proliferative and metastatic abilities of breast cancer.

### 3.3 Diagnostic efficacy of plasma cell‐free hsa_circ_0008673

**Figure 3:**
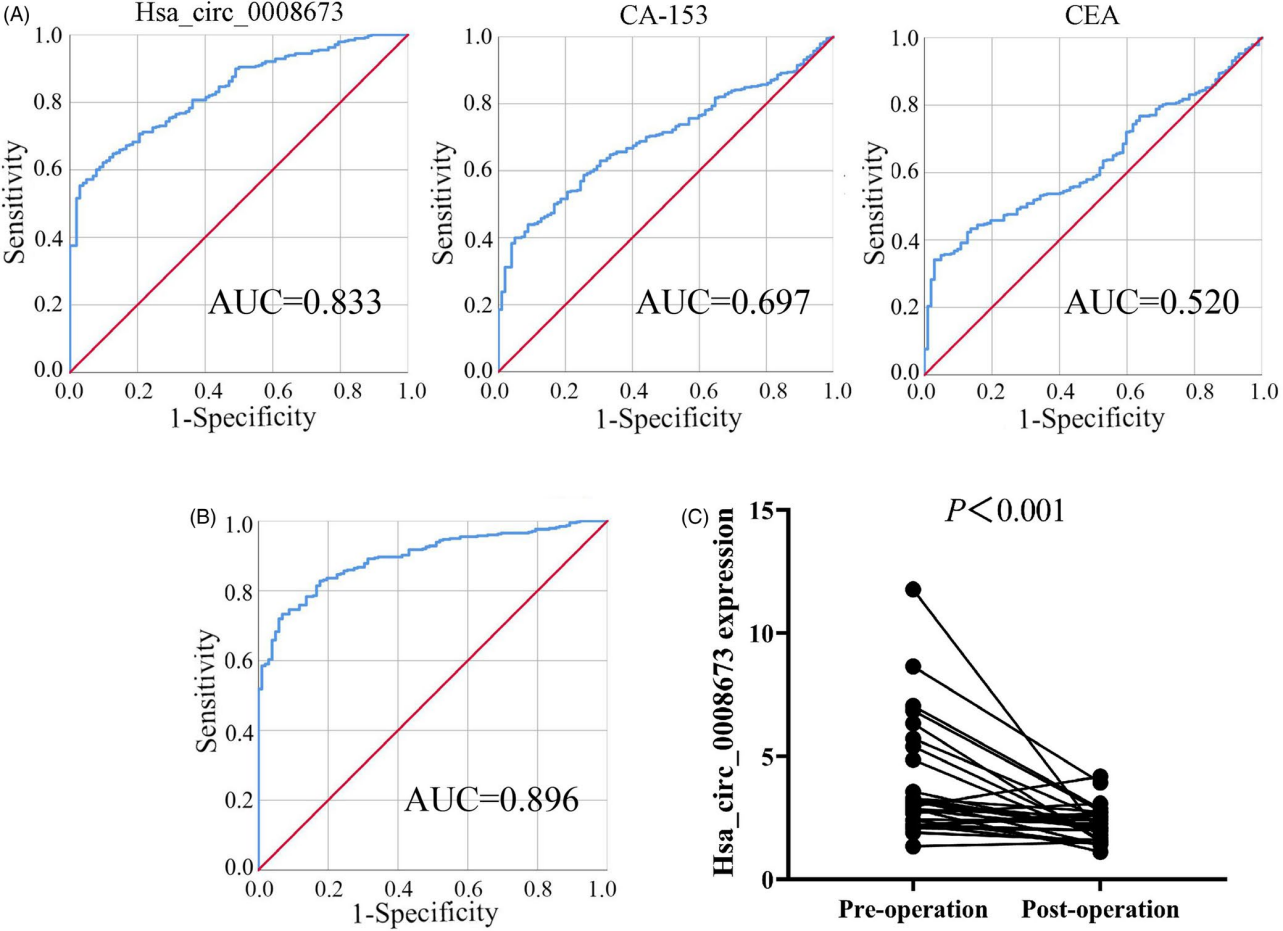
ROC curve analysis based upon plasma cell‐free hsa_circ_0008673 to evaluate the diagnostic value. A, Diagnostic values of plasma cell‐free hsa_circ_0008673, CA153, and CEA. B, A multivariable logistic regression model was performed to evaluate the combined values of plasma hsa_circ_0008673, CEA, and CA153. The combined index showed greater predictability than hsa_circ_0008673 separate measurements. C, Comparison of plasma hsa_circ_0008673 level between pre‐ and postoperative samples from 30 breast cancer patients. The hsa_circ_0008673 expression level was significantly reduced in postoperative plasma compared to preoperative plasma

Since the expression of hsa_circ_0008673 was increased in breast cancer samples and was correlated with malignant biological behaviors, we further examined the diagnostic efficacy of plasma cell‐free hsa_circ_0008673. As shown in Figure 3A, ROC curve analyses presented that plasma cell‐free hsa_circ_0008673 could serve as a forceful noninvasive biomarker for distinguishing breast cancer patients from healthy controls with an AUC value of 0.833 (95% CI = 0.795‐0.871). At the cutoff value of 1.380, the specificity was 97.10% and the sensitivity was 55.00%. We also evaluated the diagnostic value of CA153 and CEA since both of them were considered as traditional screening and subsequent management biomarkers of breast cancer patients.^18^ CA153 manifested an AUC value of 0.697 with a specificity of 95.16% and a sensitivity of 38.62%, and the AUC value of CEA was 0.520 with the specificity of 97.14% and sensitivity of 34.13%. A multivariable logistic regression model was further performed to evaluate the combined values of plasma hsa_circ_0008673, CEA, and CA153, the combined index showed even greater predictability than hsa_circ_0008673 alone, with the AUC value of 0.896, the specificity of 93.12%, and the sensitivity of 73.33% (Figure 3B). These results indicate that plasma hsa_circ_0008673 could serve as a screening biomarker for BC.

Moreover, to evaluate the tumor monitoring values of plasma hsa_circ_0008673 in breast cancer patients, qRT‐PCR method was used to measure the expression level of hsa_circ_0008673 in 30 paired pre‐ and postoperative plasma samples from breast cancer patients. The hsa_circ_0008673 expression level was found to be significantly reduced in postoperative plasma compared to that in preoperative plasma (Figure 3C, *P* < .001). This result suggests that the levels of hsa_circ_0008673 in plasma might reflect the expression in tumor.

### 3.4 Association of plasma cell‐free hsa_circ_0008673 expression with the prognosis of breast cancer patients

**Figure 4:**
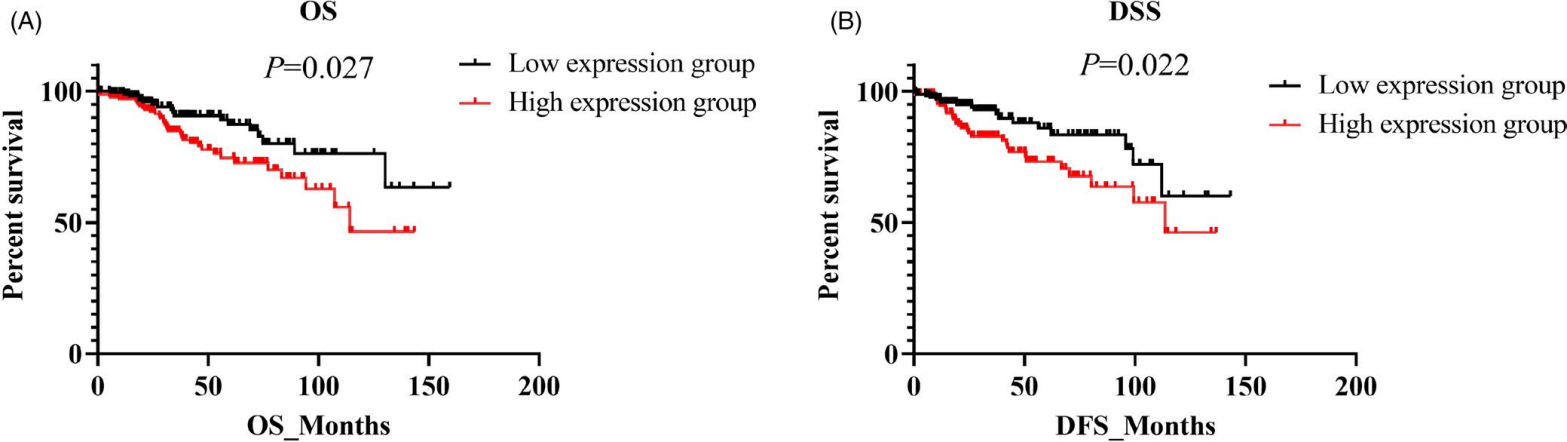
Kaplan‐Meier survival analysis of breast cancer patients according to plasma cell‐free hsa_circ_0008673 expression. A, The OS rate of breast cancer patients according to cell‐free hsa_circ_0008673 expression. B, The DSS rate of breast cancer patients according to cell‐free hsa_circ_0008673 expression. The log‐rank test was used to compare the differences between subgroups

To further evaluate the clinical significance of cell‐free hsa_circ_0008673, the association between hsa_circ_0008673 and OS (overall survival) and DSS (disease‐specific survival) was analyzed. As shown in Figure 4, the Kaplan‐Meier survival curves manifested that breast cancer patients with high plasma cell‐free hsa_circ_0008673 expression had significantly worse OS (Figure 4A) and DSS (Figure 4B) than those with low hsa_circ_0008673 expression, indicating that cell‐free hsa_circ_0008673 was a potential biomarker for predicting the prognosis of breast cancer.

### 3.5 Factors affecting the prognoses of breast cancer patients

Univariate Cox's analysis displayed that hsa_circ_0008673 (high vs low) was associated with poorer prognoses for both OS (HR = 1.900, *P* = .027) and DSS (HR = 1.989, *P* = .022). Higher pathological grade, larger tumor size, positive lymph node status, distant metastasis and no radiotherapy were also associated with poorer OS and DSS of breast cancer patients. ER, PR, and chemotherapy were only significant when OS was analyzed (Table 2). Significant factors were further analyzed by multivariate Cox's proportional hazard regression analysis, which revealed that hsa_circ_0008673 (high vs low) was an independent predictor of poorer OS (HR = 1.742, *P* = .047) and DSS (HR = 1.949, *P* = .029). Pathological grade III (HR = 5.096, *P* = .003), tumor size of T3 (HR = 2.585, *P* = .034), positive lymph node (HR = 1.903, *P* = .042), and distant metastasis (HR = 2.383, *P* = .013) independently predicted poorer OS. For DSS, tumor size of T3 (HR = 3.323, *P* = .019) and distant metastasis (HR = 2.195, *P* = .034) were also considered as independently factors of poorer prognosis (Table 3).

**Table 2:** Univariate Cox's proportional hazard regression analysis of factors affecting OS and DSS

	OS		DSS	
	HR (95% CI)	*P* value	HR (95% CI)	*P* value
CircRNA expression				
Low	REF	‐	REF	‐
High	1.900 (1.108‐3.257)	.027	1.989 (1.154‐3.430)	.022
Age at diagnosis, y				
<60	REF	‐	REF	‐
≧60	1.151 (0.666‐1.988)	.615	0.862 (0.467‐1.591)	.635
Pathological grade				
Grade I	REF	‐	REF	‐
Grade II	2.237 (0.945‐5.294)	.067	0.979 (0.486‐1.962)	.953
Grade III	4.862 (1.729‐13.677)	0.003	2.412 (1.235‐3.065)	0.019
Tumor size				
T1	REF	‐	REF	‐
T2	1.203 (0.625‐2.316)	.580	1.454 (0.630‐3.354)	.381
T3	3.565 (1.546‐8.223)	0.003	4.178 (1.643‐10.625)	0.003
Node stage				
Negative	REF	‐	REF	‐
Positive	2.189 (1.237‐3.874)	.007	1.868 (1.013‐3.442)	.045
Metastasis stage				
Negative	REF	‐	REF	‐
Positive	2.745 (1.508‐4.995)	.001	2.617 (1.311‐5.221)	.006
ER status			‐	‐
Negative	REF	‐	REF	‐
Positive	0.562 (0.320‐0.986)	.044	0.616 (0.330‐1.150)	.128
PR status				
Negative	REF	‐	REF	‐
Positive	0.574 (0.333‐0.989)	.045	0.826 (0.449‐1.520)	.539
Her‐2 status				
Negative	REF	‐	REF	‐
Positive	1.038 (0.507‐2.127)	.919	1.209 (0.539‐2.713)	.645
Radiotherapy				
No	REF	‐	REF	‐
Yes	0.411 (0.184‐0.841)	.033	0.187 (0.064‐0.683)	.005
Chemotherapy				
No	REF	‐	REF	‐
Yes	0.531 (0.451‐0.873)	.039	0.628 (0.346‐1.142)	.127

**Table 3:** Multivariate Cox's proportional hazard regression analysis of factors affecting OS and DSS

	OS		DSS	
	HR (95% CI)	*P* value	HR (95% CI)	*P* value
CircRNA expression				
Low	REF	‐	REF	‐
High	1.742 (1.016‐3.547)	.047	1.949 (1.115‐3.842)	.029
Pathological grade				
Grade I	REF	‐	REF	‐
Grade II	1.644 (0.673‐4.016)	.275	0.909 (0.450‐1.836)	.790
Grade III	5.096 (1.754‐14.808)	.003	1.853 (0.631‐5.445)	.262
Tumor size				
T1	REF	‐	REF	‐
T2	0.794 (0.394‐1.600)	.519	1.417 (0.607‐3.306)	.420
T3	2.585 (1.077‐6.207)	.034	3.323 (1.219‐9.059)	.019
Node stage				
Negative	REF	‐	REF	‐
Positive	1.903 (1.024‐3.536)	.042	1.872 (0.998‐3.509)	.051
Metastasis stage				
Negative	REF	‐	REF	‐
Positive	2.383 (1.204‐4.715)	.013	2.195 (1.062‐4.534)	.034
ER status			‐	‐
Negative	REF	‐	‐	‐
Positive	0.946 (0.400‐2.238)	.899	‐	‐
PR status				
Negative	REF	‐	‐	‐
Positive	0.533 (0.230‐1.238)	.144	‐	‐
Radiotherapy				
No	REF	‐	REF	‐
Yes	1.047 (0.570‐1.924)	.883	0.670 (0.360‐1.244)	.204
Chemotherapy				
No	REF	‐	‐	‐
Yes	0.900 (0.500‐1.622)	.726	‐	‐

### 3.6 Knockdown of hsa_circ_0008673 suppressed the proliferation and metastasis of breast cancer cells

**Figure 5:**
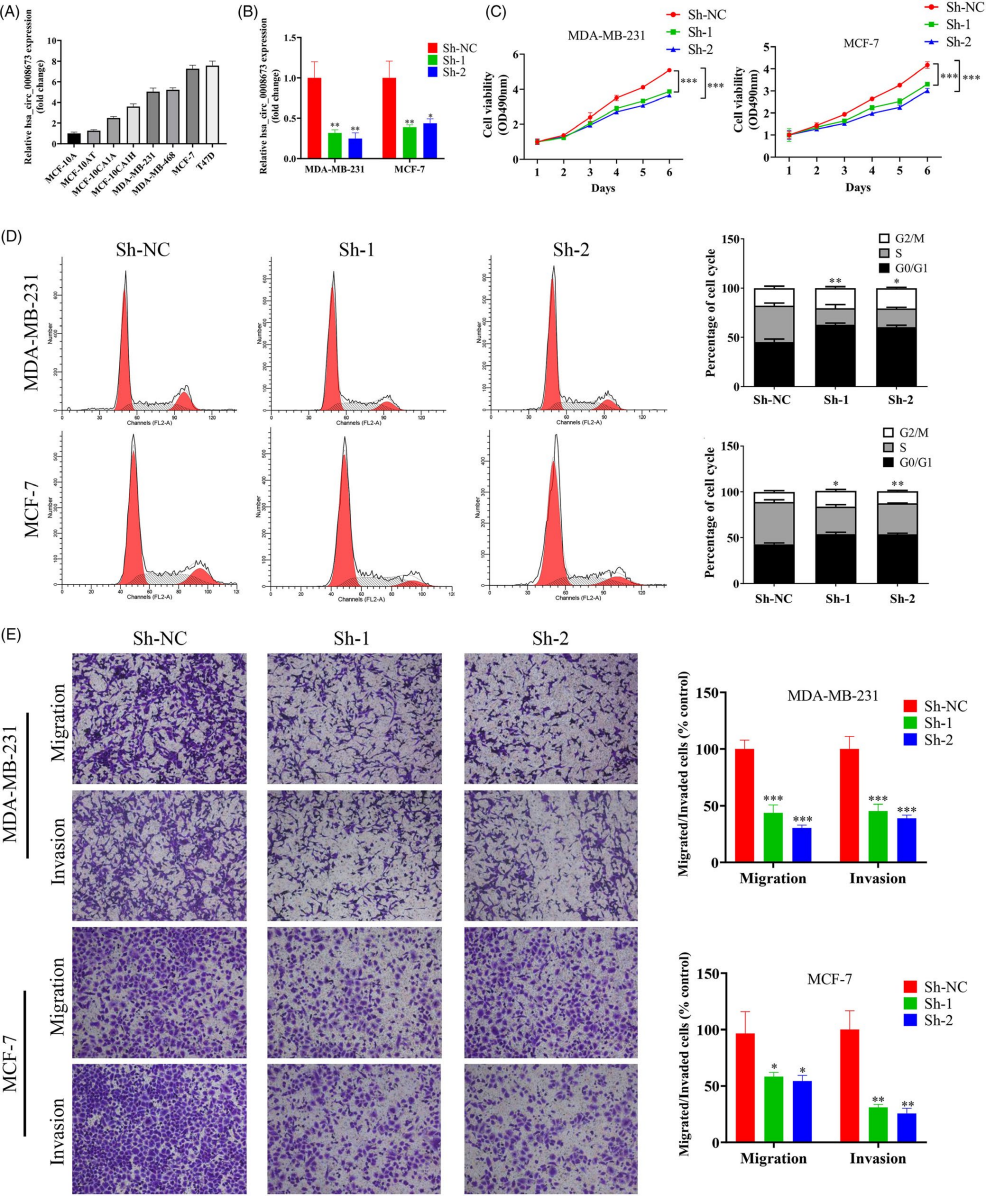
Effect of hsa_circ_0008673 knockdown on the proliferation and metastasis of breast cancer cells. A, The expression of hsa_circ_0008673 in breast cancer cell lines. B, The efficiency of hsa_circ_0008673 knockdown. C, Effect of hsa_circ_0008673 knockdown on the proliferation of breast cancer cells based on MTT assay. D, Flow cytometry verified the cell cycle of MDA‐MB‐231 and MCF‐7 cells. E, Transwell assay was performed to evaluate effects of hsa_circ_0008673 knockdown on cell migration and invasion. *P < .05, **P < .01, and ***P < .001

To further investigate the mechanism of hsa_circ_0008673 in breast cancer, hsa_circ_0008673 relative expressions in breast cancer cell lines and normal breast epithelial cell line were detected by qPCR. As shown in Figure 5A, we found that hsa_circ_0008673 was up‐regulated in breast cancer cell lines and down‐regulated in normal breast epithelial cell line (MCF‐10A), which indicated that hsa_circ_0008673 might serve as a potential oncogene in breast cancer progression. The expression of hsa_circ_0008673 was then silenced in both MDA‐MB‐231 and MCF‐7 cell lines for further analyzing (Figure 5B). The proliferation abilities of MDA‐MB‐231 and MCF‐7 cell lines were examined by MTT assay (Figure 4C), and cell cycle changes were detected by flow cytometric analysis of PI histograms (Figure 4D). We found that knockdown of hsa_circ_0008673 significantly inhibited the proliferation of both cell lines via inducing G1 phase cell cycle arrest. Transwell assay also presented that silencing of hsa_circ_0008673 suppressed cell migration and invasion of both MDA‐MB‐231 and MCF‐7 cell lines (Figure 4E). In conclusion, our results suggest that knockdown of hsa_circ_0008673 suppresses the proliferation and metastasis of breast cancer cells.

## 4 DISCUSSION

The identification of molecular markers in body fluids (eg, sera and urine), which can be used as noninvasive diagnostic, prognostic, and surveillance markers in cancer management, is one of the most ambitious challenges in oncologic research.^19^ To date, the increased interest on noninvasive biomarkers, allowed by using novel methodologies (such as next‐generation sequencing, single‐cell sequencing approaches, and digital PCR), has greatly improved the translational potential of researches into clinical application.^20^, ^21^ Recently, circRNAs have been considered as greatly potential biomarkers in many kinds of tumors due to their stability in body fluids,^11^ such as lung cancer,^12^, ^22^ pancreatic cancer,^23^ and gastric cancer.^24^

CircRNAs are a novel class of RNAs with O‐shaped closed structure existing in the living cells. Unlike traditional linear RNA molecules, circRNAs are resistant to degradation by exonuclease and RNases because there are no 5′ end, 3′ end, or even poly(A) tail.^25^ Hence, circRNAs can stably exist in cells for a long period of time, which could serve as favorable biomarkers. Furthermore, circRNA molecules in human cells are 10‐fold more numerous than the number of homogenetic linear isomer RNA molecules.^26^ CircRNA molecules have highly conserved sequences, a stable existence, and tissue‐specific expression. Also, circRNAs have been demonstrated to regulate gene expression in post‐transcriptional ways.^27^ For example, circRNAs can act as microRNA (miRNA) sponges. Li et al reported that circ‐ITCH competitively sponged miR‐7, miR‐17, and miR‐214, leading to higher expression of the ITCH gene. The ITCH gene product has been shown to inhibit Dvl2 phosphorylation and, furthermore, to inhibit the Wnt signaling pathway, which prevents tumorigenesis in the esophagus.^28^ In addition, many differentially expressed circRNAs have been investigated in tissue, blood,^29^ saliva,^30^ and other bodily fluid samples,^31^ suggesting that circRNA molecules can serve as biomarkers in many diseases. CircRNAs, together with other known biomarkers, may be able to improve the accuracy of specificity of diagnosis in certain diseases. However, most of studies mainly focused on the potential functions, whereas their clinical diagnostic value remains largely unknown specifically in breast cancer.

In our study, we firstly found hsa_circ_0008673 was remarkably up‐regulated in 6 breast cancer samples compared with 6 normal controls based on microarray and qRT‐PCR (Figure 1A‐C), which was further verified in 378 breast cancer and 102 normal samples (*P* ˂ .001) by quantitative qRT‐PCR assays (Figure 1D). Clinicopathological features showed that up‐expression of hsa_circ_0008673 level was positively associated with larger tumor size, distant metastasis, positive ER status, and positive PR status, indicating hsa_circ_0008673 might contribute to malignant behaviors of breast cancer (Figure 2). Furthermore, we evaluated whether hsa_circ_0008673 could serve as a valuable biomarker for the diagnosis of breast cancer. As shown in Figure 3A, hsa_circ_0008673 could distinguish breast cancer patients from normal controls, which proved its diagnostic value. Indeed, the AUC and the specificity of hsa_circ_0008673 were much higher than those of the conventional tumor markers such as CA153 and CEA. Further, qRT‐PCR showed that the level of hsa_circ_0008673 in plasma might reflect the expression in tumor (Figure 3C). Based on the Kaplan‐Meier method, we showed that hsa_circ_0008673 could predict the prognosis of both OS and DSS in breast cancer patients, in which high expression of hsa_circ_0008673 predicted poorer prognosis (Figure 4).

Also in this study, we took further steps to ectopically silence hsa_circ_0008673 in breast cancer cells and then evaluate the functional role of hsa_circ_0008673 in cancer malignant behaviors. Through several in vitro assays, we found that hsa_circ_0008673 inhibition significantly suppressed both tumor cell proliferation via inducing G1 phase cell cycle arrest and metastatic abilities (Figure 5). These data suggest that hsa_circ_0008673 could influence the malignant behaviors of breast cancer cells.

In conclusion, our research found a novel biomarker, which was positively correlated with the malignant clinical characteristic of patients, and could distinguish breast cancer from normal control and predict the prognosis of both OS and DSS. Moreover, we identified that hsa_circ_0008673 was an oncogene, which contributed to the proliferation and metastasis of breast cancer cells. This study sheds lights on understanding the mechanisms of disease‐associated circRNAs and improving the diagnosis and prevention of circRNA‐associated diseases.

## CONFLICT OF INTEREST

The authors declare no conflict of interest.

## Supporting information

Table S1

## Data Availability

The data used to support the findings of this study are available from the corresponding author upon request.
