# Supplementary material for: Identification of plasma hsa_circ_0008673 expression as a potential biomarker and tumor regulator of breast cancer
Source: J Clin Lab Anal. 2020 Aug 18;34(9):e23393. doi: 10.1002/jcla.23393 (PMC7521290; doi:10.1002/jcla.23393)
Supplement: Supplementary file 1 — Table S1 [file JCLA-34-e23393-s001.doc]

| **circRNA ID** | **Genomic position** | **Mature length** | **Gene symbol** | **Regulation** | **Fold change**  **(log2)** |
| --- | --- | --- | --- | --- | --- |
| hsa_circ_0008673 | chr17: 41247862-41276132 | 689bp | BRCA1 | up | 4.01 |
| hsa_circ_0008500 | chr3:196831773-196846401 | 381bp | DLG1 | up | 3.56 |
| hsa_circ_0005260 | chr5:14316621-14336836 | 546bp | TRIO | up | 3.27 |
| hsa_circ_0003423 | chr18: 56601664-56621031 | 804bp | ZNF532 | up | 3.10 |
| hsa_circ_0119881 | chr2: 36615114-36623930 | 8816bp | CRIM1 | up | 2.91 |
| hsa_circ_0000987 | chr2: 30748452-30756180 | 368bp | LCLAT1 | up | 2.91 |
| hsa_circ_0007386 | chr2: 36668400-36669878 | 364bp | CRIM1 | up | 2.69 |
| hsa_circ_0000091 | chr1: 92798947-92846430 | 383bp | RPAP2 | up | 2.69 |
| hsa_circ_0016601 | chr1: 225140371-225195246 | 1140bp | DNAH14 | up | 2.69 |
| hsa_circ_0008549 | chr3: 31917924-31921322 | 256bp | OSBPL10 | up | 2.69 |
| hsa_circ_0000826 | chr18: 9182379-9221997 | 994bp | ANKRD12 | down | -2.22 |
| hsa_circ_0072697 | chr5: 64863339-64868113 | 773bp | PPWD1 | down | -2.22 |
| hsa_circ_0004587 | chr6: 158994451-159010814 | 484bp | TMEM181 | down | -2.22 |
| hsa_circ_0000471 | chr13: 33091993-33101669 | 388bp | N4BP2L2 | down | -2.22 |
| hsa_circ_0007786 | chr8: 141840570-141900868 | 680bp | PTK2 | down | -2.22 |
| hsa_circ_0001417 | chr4: 73950965-73958017 | 1832bp | ANKRD17 | down | -2.48 |
| hsa_circ_0005982 | chr8: 141828375-141900868 | 899bp | PTK2 | down | -2.48 |
| hsa_circ_0001566 | chr5: 179688683-179707608 | 497bp | MAPK9 | down | -2.48 |
| hsa_circ_0003823 | chr3: 138289159-138291826 | 522bp | CEP70 | down | -2.48 |
| hsa_circ_0007683 | chr12: 27149674-27152609 | 272bp | TM7SF3 | down | -3.07 |

**Table S.1.** The detailed information about the top ten up- and down-regulated circRNAs between breast cancer group and control group
